# First Lines of Defence

**Published:** 1892-09-03

**Authors:** 


### The Hospital

AN INSTITUTIONAL JOURNAL OF

### Science, Medicine, Nursing, and Philanthropy.

*For Hospitals, Asylums, Infirmaries, Dispensaries, Medical Practitioners, Students, Nurses, and the Charitable Public.*

VOL. XII.—No. 310.] [SEPT. 3, 1892.

### FIRST LINES OF DEFENCE.

THERE is no doubt the enemy of cholera is at our gates, though it is by no means certain that he will be able to effect a lodgment in our midst. Panic is, of course, to be avoided; but so also is dull indifference. The rich are in less danger than the poor; but it does not therefore follow that they are in no danger at all. On the contrary, it is quite certain, if the enemy once get well within our gates, that he will take his toll of the rich as well as of the poor.

The present is a time when friendly feeling and intelligent co-operation are among the most cardinal of virtues. The natural leaders of the people must not hesitate to take the lead with boldness; nor must they grudge the spending of money freely. What are a few thousands of pounds, or even a few hundred thousands, compared with the preservation of the life and of the morale of our people? On the other hand, the poor and the ignorant must try to trust those who are better instructed than themselves, and to follow their lead. For, after all, the risks of the poor are tenfold greater than those of the rich. It is certain that the official leaders, and the better classes generally, will do all that in them lies to avert impending calamity. But, without the entire sympathy and co-operation of all classes, how very little it is that they can really do! The most important of all the first lines of defence which our own or any other nation can construct is the hearty goodwill of all classes of the people towards one another, and a loyal determination to stand shoulder to shoulder, and to promptly obey orders against the common enemy.

We are not forgetting the sentiments just expressed when we venture to hint a little surprise that emigrant and other vessels from Hamburg and Antwerp have been permitted to come so freely into our ports and to disembark so many doubtful and dangerous persons on our shores. It is manifest to all men, who are in the habit of considering, that the commerce of the world's market and the world's factory, as England still to so large an extent is, cannot be lightly interfered with. But, on the other hand, we must lay our account with the fact that physiology and disease are absolutely indifferent to commercial considerations. This is especially the case with diseases of an epidemic character. They rush hither and thither, they seize upon the useful and useless alike; and they slay without discrimination and without hesitation the statesman on whose word a nation may depend, or the employer of thousands, whose death may bring manifold starvation in its train.

This undiscriminating behaviour of epidemic disease is apt to be overlooked by men and leaders of the people whose lives are devoted to the conducting of great enterprises. But it is obvious that the physician must take note of the blind and unhalting march of the enemy. It is as much the doctor's duty to insist upon defensive out-works, and upon the necessity for keeping the foe at long arm's length, as it is the interest of the man of business to protest against unnecessary interference with commerce. Nay, if we must choose between a temporary suspension of business, and the wholesale decimation of our people by so dread a disease as cholera, who can hesitate what choice to make? If the physician failed to warn his fellow-countrymen of such notable danger, he would be a watch-dog that failed to bark when the thief was at the door; and a sentry who threw down his arms at the first approach of the enemy. The practical conclusion of this reasoning is, that we must not hesitate to prevent suspicious aliens from landing on our shores, or even from entering our ports, if need should arise. For after all, it is our own people that our leaders are bound in the first place to protect. The leaders of other nations cannot, with any justice, ask us to take charge of their helpless and their sick. It may sound selfish and provincial to say that "charity begins at home." But for all that, charity does begin at home; and this is a principle upon which our leaders must not hesitate to act.

Probably all of us can see the duty of responsible public officials very clearly. It is of as great, or even of greater importance, that we should see our own duty. Everybody knows that a fire burns most swiftly and destructively when it seizes upon vast masses of combustible materials. Without such combustible materials the fire, indeed, cannot burn at all. Whilst we insist upon the duty of our public officials to beat out the approaching flames while yet they are a great way off, we must equally, and even more, insist upon the duty of families and individuals to remove all combustible materials with the utmost promptitude and thoroughness. When we consider all the admirable instructions given in our morning and evening newspapers from day to to day, it is difficult to believe that any grown-up person can be found who does not understand what ought to be done in private houses to render them practically proof against cholera. Filth is not such a delightful companion in a house that any of us need regard it as a deposit too sacred to be touched. It would not be a very tremendous thing to ask people who live in small houses to have every room cleansed and limewashed, every drain and closet swilled with water and purified by disinfectants. No doubt many people are too poor to provide themselves with brushes, lime, permanganate of potash, and the like. But it is as certain as certain can be that if many of them showed a disposition thus to take upon themselves the work of the preservation of their own lives, the public sanitary services would be only too glad to supply them with all needed material free of cost.

It is necessary for all people to keep in mind three or four great facts which constitute the leading features of sanitation. They are pure air, pure water, wholesome food, and the destruction or deportation of filth and excrementitious matters. In order to purify the air, rubbish of all kinds, both in yards and in houses, should either be burnt, or given up to the dustman to be carted away. For purification of the water, all that is needed is that it be well boiled. To sweeten small houses, let the floors be well scrubbed, the wall papers torn off and burnt, and the walls lime-washed. Wholesome food may be insured by living largely on bread, and by purchasing only such small quantities of meat, vegetables, and fruit as can be procured perfectly fresh. For the drainage, as we have said, disinfectants must be used in abundance; and these, we have no doubt, will be supplied by the public authorities wherever it is necessary.

The industrial classes should consider this fact, that the rich, who are in little danger, will take all the precautions here indicated, and far more; whilst the poor, who may be said, like the Prophet Balaam, to be standing in the invisible presence of the Angel of Death, will hardly lift a finger, even for the saving of their own lives. Of two things, one or the other will happen; either we shall rise as a people to a great emergency, and keep the enemy outside our gates: or we shall be compelled by the agonies of the dying thousands in our midst to rise to a much greater emergency in order to turn out an enemy who has entered within our gates and taken deadly possession of our homes.

